# What helps and what hinders antidepressant discontinuation? Qualitative analysis of patients’ experiences and expectations

**DOI:** 10.3399/BJGP.2023.0020

**Published:** 2024-05-28

**Authors:** Carina Meißner, Ann-Katrin Meyrose, Yvonne Nestoriuc

**Affiliations:** Clinical Psychology and Psychotherapy, Helmut-Schmidt-University/University of the Federal Armed Forces Hamburg; Institute of Systems Neuroscience, University-Medical Center Hamburg-Eppendorf, Hamburg.; Clinical Psychology and Psychotherapy, Helmut-Schmidt-University/University of the Federal Armed Forces Hamburg; Department of Child and Adolescent Psychiatry, Psychotherapy, and Psychosomatics, University-Medical Center Hamburg-Eppendorf, Hamburg.; Clinical Psychology and Psychotherapy, Helmut-Schmidt-University/University of the Federal Armed Forces Hamburg; Institute of Systems Neuroscience, University-Medical Center Hamburg-Eppendorf, Hamburg.

**Keywords:** Major depressive disorder, primary health care, psychotropic drugs, Germany

## Abstract

**Background:**

Many patients with depressive disorders use antidepressants longer than clinically indicated. Long-term use of antidepressants is associated with high individual and societal costs. Patients often perceive antidepressant discontinuation as challenging.

**Aim:**

To understand patients’ expectations about discontinuation, to document their experiences with long-term use and discontinuation, and to identify factors that can help or hinder discontinuation.

**Design and setting:**

Qualitative study using semi-structured interviews via telephone with adult patients in Germany.

**Method:**

We interviewed 32 patients with remitted major depressive disorder and long-term antidepressant use. We analysed transcripts with content analysis aided by MAXQDA to derive thematic categories.

**Results:**

Patients expected to eliminate side effects or regain independence after discontinuation. Such positive expectations were perceived as facilitators and motivated patients’ wish to discontinue antidepressants. However, patients also had negative expectations such as recurrence or discontinuation symptoms. Patients’ negative expectations were often fuelled by previous negative experiences, which persisted despite a wish to stop antidepressants, and hindered discontinuation. Most patients perceived antidepressants as being effective, but experienced side effects and further problems. Patients felt inadequately informed about treatment duration and methods for discontinuation. Further barriers and facilitators included a stable environment, availability of support, and treatment information.

**Conclusion:**

Patients prefer to discontinue antidepressants within structured frameworks that provide information and support. Identified facilitators and barriers may help optimise appropriate use and discontinuation of antidepressants in routine practice. Promoting functional expectations and specifying individualised approaches to minimise dysfunctional expectations, adapted to patients’ previous experiences, appear to be especially important.

## Introduction

Between 30% and 50% of patients with long-term antidepressant use for treatment of major depressive disorder (MDD) may consider antidepressant discontinuation.^1^^–^3 Treatment guidelines recommend continuing antidepressant use for at least 6 months to treat a single moderate-to-severe episode.^4^^–^6 In case of recurring episodes, treatment guidelines advise antidepressant use for ≥2 years.^4^^–^7 Continued long-term use is only recommended in case of high recurrence rates or chronic depression.^4^^–^7 Following sustained remission, most patients are expected to discontinue antidepressants.^4^^–^7

Long-term use of antidepressants is associated with high societal expenses and individual burden. In Germany, approximately 7% of the population use antidepressants,^8^ with antidepressant prescriptions resulting in net costs of over €640 million in 2020 alone.^9^ Antidepressant use is regularly accompanied by burdensome side effects.^10^^,^^11^ These include sleep disturbances, dry mouth, sexual dysfunction, and psychological dependency,^12^^–^15 and often persist during long-term use.^14^

The efficacy and tolerability of antidepressants are influenced by patients’ expectations.^16^^–^19 Patients’ positive and negative expectations concerning their health state can improve or impair their health outcomes through placebo and nocebo effects.^20^ Rief *et al*^18^^,^^19^ investigated these effects in antidepressant trials and found significantly reduced depressive symptoms under placebo conditions, as well as nocebo-induced side effects. These findings highlight the need to also consider the role of expectations in antidepressant discontinuation.^21^^,^^22^

Antidepressant discontinuation is frequently accompanied by adverse discontinuation symptoms.^23^ These include flu-like syndromes and sensory, gastrointestinal, cognitive, or other disturbances.^24^^,^^25^ Some patients appear to be at increased risk for recurrence of MDD following antidepressant discontinuation.^26^^,^^27^ Certain discontinuation symptoms resemble depressive symptoms, including irritability or suicidal ideation. This resemblance confounds recurrence assessments in discontinuation trials,^1^ and complicates differential diagnosis.^28^^,^^29^ Misdiagnosing discontinuation symptoms as signs of recurrence can result in inappropriate treatment decisions and contribute to unnecessary long-term use of antidepressants.^28^

**Table:** How this fits in

Long-term antidepressant use is increasing, including among those patients who may consider discontinuation. In this study, patients with remitted major depressive disorder and long-term antidepressant use reported negative expectations about discontinuation. These expectations were partly shaped by their previous negative experiences, which persisted despite a wish to stop antidepressants, and hindered discontinuation. The findings of this study highlight patients’ need for information about treatment discontinuation, and professional support and structure throughout discontinuation, while taking into account their individual expectations and previous experiences.

Many patients wish to stop using antidepressants, but perceive antidepressant discontinuation as challenging.^30^^,^^31^ Qualitative research in Western countries has investigated the barriers to and facilitators of discontinuation.^10^^,^^32^^–^34 Fear of recurrence or discontinuation symptoms can hinder discontinuation.^35^^,^^36^ Previous negative experiences can fuel such fears.^33^ Professional guidance can help patients make an informed decision about discontinuation and to feel assured in case of symptom deterioration after discontinuation. However, patients report inadequate availability of support and information for a safe discontinuation process.^28^^,^^34^^,^^35^^,^^37^

We conducted semi-structured interviews with patients with remitted MDD and long-term antidepressant use to understand their expectations about antidepressant discontinuation, to document their experiences with long-term use and discontinuation, and to identify factors that can help or hinder discontinuation. To our knowledge, this is the first qualitative study to examine patients’ expectations about discontinuation, and the first in Germany to examine patients’ experiences and facilitators and barriers.

## Method

We conducted this study within a mixed-methods framework and in preparation of a randomised controlled trial (ClinicalTrials.gov identifier: NCT05191277). The conception and conduct of the study were in accordance with consolidated criteria for reporting qualitative research.^38^ Reporting followed Standards for Reporting Qualitative Research.^39^

### Participants and recruitment

We conducted semi-structured interviews with 32 adult patients with remitted MDD and long-term antidepressant use in primary health care. In Germany, antidepressants are mainly prescribed by GPs, psychiatrists, and neurologists. German citizens must take out health insurance and medical care is financed by the resulting social security funds. German citizens therefore have free access to healthcare services, and are able to self-refer and access specialist services.^40^

We approached potential participants between June and November 2020. Recruitment included purposive and convenience sampling strategies throughout Germany via primary healthcare professionals, self-help groups, leaflets, social media, and online forums. Interested patients could contact the study team or access a study webpage for further information. As an incentive, patients had the chance to win one of seven €15 vouchers and received a clinical assessment concerning their possibility to discontinue antidepressants (see Supplementary Information S1 for details) according to German guideline recommendations.^4^ After participants had read the study information and provided informed consent, their eligibility was assessed using an online screening questionnaire (see Supplementary Information S2 for details). Eligibility criteria are outlined in Box 1. If excluded from the study, participants received information about the reasons for exclusion and an overview of support services. Eligible participants were automatically forwarded to the quantitative online survey. Subsequently, participants indicated their contact information and qualitative interviews were arranged.

**Box 1.** Eligibility criteria for interview study

**Inclusion criteria**
Adult patients (aged ≥18 years) with remitted major depressive disorder, single or recurrent, confirmed by a Beck Depression Inventory II (BDI-II) sum score ≤19.^41^ Remission was defined as the presence of at most mild residual depressive symptoms, confirmed by a BDI-II sum score ≤19.^41^ According to German treatment guidelines,^4^ a BDI-II sum score ≤13 indicates full remission. BDI-II sum scores ≤19 fall below moderate depressive symptoms, that is, the guideline-recommended cut- off for antidepressant treatment,^4^ and indicate partial remission. Long-term use of a selective serotonin reuptake inhibitor (citalopram, escitalopram, fluoxetine, paroxetine, or sertraline) or a selective serotonin norepinephrine reuptake inhibitor (venlafaxine or duloxetine), that is, for at least 6 months in case of a single depressive episode, and at least 24 months in case of recurrence (constant dose within the last month). Reported consideration of or wish to discontinue.
**Exclusion criteria** Acute or chronic somatic illness. Any history of bipolar or psychotic disorder. Substance abuse or addiction within the last 24 months. Insufficient German language proficiency. No informed consent.

### Data generation

We conducted interviews in German via telephone using a pilot-tested, semi-structured interview guide with open-ended questions (see Supplementary Information S3 for details) developed by the researchers. One researcher facilitated interviews and was the contact person for patients. Two researchers supervised psychology students, conducted, audio-recorded, and transcribed interviews verbatim. One researcher re-listened to recordings and edited transcripts. Interviews were conducted until no new relevant knowledge was obtained.

No relationship between participants and interviewers was established before study commencement. The setting for data collection varied between the university campus and private workplaces because of restrictions relating to the COVID-19 pandemic. Participants self-reported their age, gender, and medical characteristics (Table 1) as part of the quantitative online survey. Transcripts were not returned to participants. No repeat interviews were conducted. Participants could indicate whether they wished to receive a summary of the study findings.

**Table 1.** Demographic and medical characteristics of participants

**Characteristic**	**Patients with remitted MDD (*N* = 32)**			

	***n***	**%**	**Median**	**Range**
Gender				
*Female*	21	65.6		
*Male*	10	31.3		
*Non-binary*	1	3.1		

Age, years			35	18–63

MDD type				
*Recurrent*	26	81.3		
*Single episode*	6	18.8		

Antidepressant type				
*SSRIs*	20	62.5		
*Citalopram*	5	15.6		
*Escitalopram*	5	15.6		
*Fluoxetine*	5	15.6		
*Sertraline*	4	12.5		
*Paroxetine*	1	3.1		
*SNRIs*	12	37.5		
*Venlafaxine*	9	28.1		
*Duloxetine*	3	9.4		

Prescriber of antidepressant				
*Psychiatrist*	24	75.0		
*GP*	5	15.6		
*Neurologist*	2	6.3		
*Self*^a^	1	3.1		

Check-ups with prescriber^b^				
*Quarterly*	9	28.1		
*Every half-year*	9	28.1		
*Once a year*	6	18.8		
*Less than once a year*	8	25.0		

Duration of antidepressant use, years			5	1–23

Depressive symptoms (BDI-II sum)			9	2–18
*Remitted*	26	81.3		
*Partially remitted*	6	18.8		

Discontinuation wish^c^			8	1–10

Discontinuation experience^d^				
*Yes*	22	68.8		
*Positive*	2	6.3		
*Neutral*	2	6.3		
*Negative*	18	56.3		
*No*	10	31.3		

a *One patient, a physician, indicated self-prescribing the antidepressant.*

b *We cannot specify whether patients’ antidepressant use was reviewed at the check-up.*

c *Discontinuation wish was assessed with the question: ‘How much do you wish to discontinue your antidepressant at this point?’ on a scale from 0 (not at all) to 10 (very much).*

d *Patients self-reported whether they had attempted discontinuation before. If so, they also rated the experienced value of their most recent discontinuation attempt on three scales from 0 (not at all) to 10 (very much) with regard to how positive, how negative, and how neutral they experienced the discontinuation attempt. BDI-II = Beck Depression Inventory II.* *MDD = major depressive disorder. SNRI = serotonin noradrenaline reuptake inhibitor. SSRI = selective serotonin reuptake inhibitor.*

### Data analysis

The software MAXQDA (2022) was used to aid qualitative content analysis. Analysis was conducted in German and findings were translated into English. After full immersion in the data, two researchers coded all 32 transcripts with deductive, concept-driven, top-level thematic codes: positive expectations, negative expectations, experiences with antidepressant use, barriers, and facilitators. These codes directly pertained to research questions and were assigned in several cycles and in close consultation with the remaining researcher. The initial coding frame was supplemented by inductive, data-driven, top-level thematic codes: general experiences with antidepressants, and experiences with discontinuation. Subsequently, inductive, data-driven, sub-level thematic codes were developed via focused case summaries, which summarise all relevant passages from the perspective of the research question per participant, and assigned in several cycles.^42^ After 13 transcripts, no further sub-level codes emerged. Two researchers independently coded the remaining transcripts. All diverging codings were discussed and full consensus reached. Code definitions were refined during multiple peer debriefing sessions. The findings were discussed and confirmed during participant checking in an audio-recorded online group discussion. The group consisted of five participants and two recovery companions. Recovery companions have first-hand experience with recovery from certain disorders and are trained to offer peer support.

## Results

### Demographic and medical characteristics

The 32 participants were aged 18–63 years, with most identifying as female (*n* = 21). Most experienced recurring episodes of MDD (*n* = 26) and were prescribed selective serotonin reuptake inhibitors (*n* = 20). Most had attempted discontinuation previously (*n* = 22) (Table 1).

**Figure 1.**
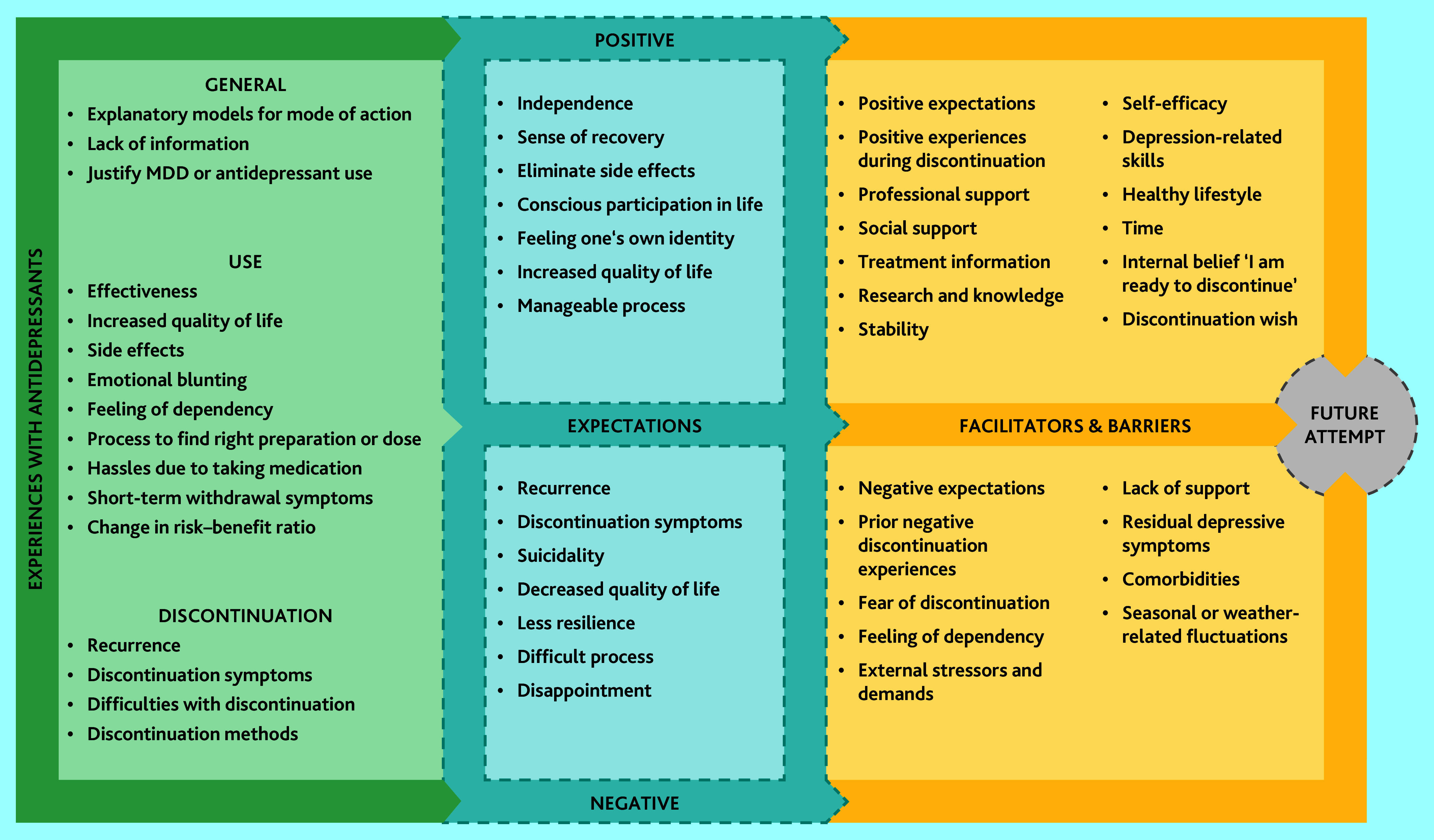
Patients’ expectations about discontinuation, their experiences with antidepressants, and barriers and facilitators. MDD = major depressive disorder.

We identified seven top-level and 52 sub-level codes. Figure 1 presents a schematic display of all identified codes. See Supplementary Box S1 for further illustrative quotations in English. For transparency, Supplementary Box S2 includes the original German quotations as well as the English translations.

### Expectations

#### Positive expectations

Patients expected to regain independence from antidepressants after discontinuation. Moreover, some patients perceived an ambivalence between their continued antidepressant use and no longer having depressive symptoms. They expected to feel a sense of recovery:

*‘To feel more independent again, perhaps, because you … make it on your own again, without medication.’*

(P27, female [F], aged 18 years [y])

*‘To complete the … process of recovery. That is, to finally restore health. This sense of closure.’*

(P29, male [M], 31y)

Another prevalent positive expectation was eliminating side effects. Among others, patients expected to eliminate *‘dry mouth’*, *‘stomach problems’*, and *‘sleep difficulties’*. Concerning psychological side effects, patients expected that discontinuation would allow a more conscious participation in life and help them feel their own identity again:

*‘That I will become more present again. Again, and again, I notice that one is very much absorbed by it* [antidepressant].*’*

(P21, F, 25y)

*‘Positive expectation: I hope to be me again.’*

(P25, M, 42y)

Some patients expected an increased quality of life resulting from discontinuation:

*‘Just to regain a bit of quality of life, so that I don’t have to take a whole battery of tablets every day … and always have the feeling that I am seriously ill.’*

(P10, M, 59y)

Ultimately, some patients expected discontinuation to be manageable and approached it with confidence, expecting to cope well with discontinuation symptoms.

#### Negative expectations

Patients expected a recurrence of depressive symptoms and occurrence of discontinuation symptoms, partly resulting from their previous experiences. Moreover, two patients expected suicidality following discontinuation:

*‘Well, that depressive symptoms will quickly deteriorate so that basically I will have to start all over again.’*

(P19, F, 34y)

*‘The typical discontinuation symptoms, which I assume will disappear with time again.’*

(P3, M, 27y)

*‘Because I’m afraid of falling back into … this hopelessness again. And then, as a result of being there again, perhaps really … take*[ing] *my own life*.*’*

(P4, M, 25y)

Some patients expected a decreased quality of life after discontinuation. This decrease was expected to result from reduced stability or worse mood. Some patients expected to be less resilient after stopping antidepressants, that is, less able to adapt to challenging life circumstances or cope with negative life events:

*‘That my quality of life in general would deteriorate again and that I would have to sacrifice … the progress I had made.’*

(P27, F, 18y)

*‘I had … tumour surgery … and I still have regular check-ups … and if there were any bad news … I’m afraid that I might somehow “fail” without any medication.’*

(P8, F, 39y)

Some patients expected discontinuation to be difficult and felt afraid, hopeless, or pessimistic about it. Some patients expected disappointment and anticipated that they would not be able to complete the discontinuation process and would feel frustrated about their failed attempt. This expectation was partly based on their previous experiences.

### Experiences

#### General

Patients illustrated heterogeneous explanatory models for the mode of action of antidepressants. Physicians did not regularly inform patients about the mode of action. Patients’ explanatory models were mostly based on their own experiences with antidepressant use, or on reports from their social environment or the internet:

*‘When my brain is overwhelmed, it sometimes results in a depression … and I would like … to be able to achieve a balance, that is, to balance the electricity in my brain.’*

(P16, F, 41y)

Patients perceived a lack of information about antidepressants. They felt inadequately informed about the effects of antidepressant use, recommended treatment duration, and methods and timing of discontinuation:

*‘Uncertainties like: “How long can I take it? How does it affect me?” Also, the lack of education or knowledge about how it even works in the long term.’*

(P26, F, 25y)

Some patients experienced the need to justify their antidepressant use or MDD.

#### Antidepressant use

For most patients, antidepressant use was an effective treatment. Some patients described how this effectiveness led to an increased quality of life, increased resilience, or an enhanced ability to enter psychotherapy. At the same time, antidepressant use was often accompanied by adverse side effects:

*‘The medication really helped me a lot, but the side effects are not to be scoffed at either.’*

(P23, F, 43y)

Side effects included physiological or psychological symptoms and difficulties. Some patients described feeling disconnected from their own emotional experience or identity. This side effect is known as emotional blunting and was emphasised as especially burdensome during participant checking. Despite such adverse side effects, the effective reduction of depressive symptoms motivated continued long-term use for many patients. Some patients, however, felt a dependency on antidepressants:

*‘Overall, there is a certain “bluntness” of feeling … I’m usually easily moved to tears and since I’ve been under medication, I’ve completely lost that.’*

(P8, F, 39y)

*‘I just feel dependent. Not physically dependent in the sense of an actual addiction, but dependent in terms of my capabilities.’*

(P31, F, 37y)

Further experiences with antidepressant use included a process to find the right preparation or dose. Patients trialled different types or dosages of antidepressants, which sometimes made patients feel exhausted, in the hope of finding the antidepressant they would respond well to. Additional causes of stress resulting from taking medication were expressed, for example, having to arrange appointments or co-payments. Moreover, some patients occasionally forgot to take their antidepressant and experienced adverse short-term withdrawal symptoms:

*‘It’s always a bit about finding a quite good ratio of effectiveness but with relatively few side effects.’*

(P3, M, 27y)

*‘I guess that I will, indeed, experience discontinuation symptoms, because I already know that if I forget to take them in the morning … I notice it by noon already.’*

(P15, F, 25y)

Most patients associated certain benefits and risks with their antidepressant use. Benefits included effectiveness and increased quality of life. Risks included side effects and hassles associated with taking antidepressants. Some patients weighed these benefits and risks, and described a change in their risk–benefit ratio over time. At some point, the perceived risks outweighed the benefits, motivating some patients to want to stop antidepressants:

*‘The risk–benefit, well, that — somehow they were really good and helped and were important, and I think that now it is just the time for me to stop.’*

(P2, F, 54y)

#### Antidepressant discontinuation

After previous discontinuation attempts, patients had experienced a recurrence of depressive symptoms. Patients also reported occurrence of discontinuation symptoms and used various synonyms, such as side effects or discontinuation phenomena, further emphasising varying degrees of knowledge about discontinuation. During past discontinuation attempts, patients faced general difficulties with discontinuation relating to personal frustration about lack of support and information. These difficulties reflected the various discontinuation methods patients used, including medically supervised or unsupervised, gradual, or abrupt dose reduction:

*‘It is much more difficult to discontinue than is somehow being suggested or than doctors even know or admit … Doctors usually say that it is easy to stop using it* [antidepressant] *after a while, but actually this is not at all the case.’*

(P23, F, 43y)

*‘I took the venlafaxine capsules, which have these little beads in them, and I sat down every evening, counted these little beads and dosed them down very slowly, always taking only five beads less.’*

(P24, F, 41y)

### Barriers and facilitators

#### Barriers

Patients perceived their negative expectations as a barrier to discontinuation. Patients with previous negative discontinuation experiences indicated that their negative experiences stopped them from attempting discontinuation again. For some, their negative experiences fuelled their negative expectations:

*‘This belief … that things will get worse then.’*

(P30, F, 24y)

*‘I can’t come off it, because I tried it once, and I had really massive discontinuation phenomena.’*

(P8, F, 39y)

Patients reported diffuse fears extending beyond recurrence or discontinuation symptoms, and perceived their fear of discontinuation as hindering discontinuation. The feeling of dependency constituted a further barrier:

*‘I’m not sure how much good it does, but it sticks to me like pigeon shit … I can’t stop it.’*

(P8, F, 39y)

External stressors and demands relating to patients’ personal or work life could also hinder discontinuation. Moreover, patients perceived a lack of support, whether social or professional, as a barrier. Finally, patients mentioned residual depressive symptoms, having comorbidities, and experiencing seasonal or weather-related fluctuations in mood as further factors hindering discontinuation.

#### Facilitators

Patients perceived their positive expectations and making positive experiences during discontinuation as facilitators:

*‘I don’t think anything will come up again, I really do believe that things will be good.’*

(P2, F, 54y)

*‘I imagine it would motivate me to realise “Gee, you’ve already made it through so and so many weeks on a low dose and you’ve been doing fine.”’*

(P19, F, 34y)

Patients indicated professional support as being central to successful discontinuation. Such professional support could be offered by physicians, psychotherapists, or qualified volunteers, such as recovery companions. Patients wanted professional support to involve a structured discontinuation framework, including regular check-ups. Having a contact person and receiving reassurance in case of uncertainties emerging during the discontinuation process was perceived as helpful. In addition, patients perceived social support as a facilitator. Forms of social support included a supportive work environment and exchange with friends, family, or other people affected:

*‘What could facilitate the discontinuation process is if you do it in close supervision with a doctor and a therapist.’*

(P15, F, 25y)

*‘Support from my immediate environment, people who are close to me.’*

(P5, non-binary, 27y)

Patients felt inadequately informed about discontinuation and requested specified treatment information early on. Patients noted a general lack of knowledge about appropriate antidepressant treatment among health professionals. They wanted more research to be conducted to generate and aggregate knowledge, so that health professionals can be trained accordingly:

*‘Being well informed … that I get information that these* [discontinuation symptoms] *are normal and that they will probably stop at some point or that I can start* [treatment] *again.’*

(P18, F, 39y)

*‘If I expect anything from society, it’s that in the future all psychiatrists will be trained on how to taper antidepressants.’*

(P25, M, 42y)

Stability in patients’ daily lives and their social environments could also help with discontinuation. Moreover, patients perceived a high sense of self-efficacy, trusting in their own capacity to cope with discontinuation, as aiding the process. Patients indicated having learned depression-related skills for managing their disease and expected these to positively affect discontinuation. Depression-related skills included mindfulness, self-care strategies, self-monitoring, and the ability to recognise signs of recurring depression and plan countermeasures. Such skills were generally attained in psychotherapy or psychoeducational groups and, for some patients, contributed to self-efficacy. A healthy lifestyle, especially engaging in physical activity and adhering to a healthy diet, was perceived as helpful. Some patients considered a healthy lifestyle as a strategy to *‘compensate’* for antidepressant use:

*‘I definitely want to take more care of myself, which means meditating, taking more walks in the fresh air. Just the things that generally help with low mood or severe depression.’*

(P28, F, 55y)

Patients described time for a slow tapering process despite day-to-day demands as a facilitator. This view was endorsed during participant checking, with the emphasis on a slow process with gradual, small, tapering steps. Some patients reported the internal belief *‘I am ready to discontinue’* and perceived it as facilitating discontinuation. This belief was accompanied by a high sense of self-efficacy and the anticipation that remission would remain stable after stopping antidepressants:

*‘When you say, “OK, you can handle it and I don’t need them any more.”’*

(P11, M, 29y)

All patients reported a discontinuation wish, albeit with varying intensity. Not all patients intended to realise this wish. For some patients, however, their strong desire to stop antidepressants was a facilitator:

*‘I think that the whole thing* [discontinuation] *won’t be that easy for me. But … I want to make it and stop taking the medication anyway.*’

(P12, M, 21y)

## Discussion

### Summary

We aimed to understand patients’ expectations about antidepressant discontinuation, to document their experiences with long-term use and discontinuation, and to identify factors that can help or hinder discontinuation. After discontinuation, patients expected to eliminate side effects, experience a sense of recovery, and regain independence. Patients perceived their positive expectations as facilitators of discontinuation. At the same time, patients also had negative expectations, such as recurrence or discontinuation symptoms. Such negative expectations were often fuelled by patients’ previous negative experiences, which persisted despite a wish to stop antidepressants, and hindered discontinuation. Antidepressant use was generally effective but associated with side effects or an unpleasant feeling of dependency. Patients felt inadequately informed about antidepressants, treatment duration, and methods and timing of discontinuation. Further barriers and facilitators included professional and social support, treatment information, and stability.

### Strengths and limitations

Our findings give an insight into patients’ expectations about antidepressant discontinuation and add a broader European dimension to the emerging body of mostly English language-based research about the difficulties associated with antidepressant use and discontinuation. Participant checking enhanced patient involvement, clinical relevance, and trustworthiness of the findings. Our sample included patients with a diverse age range, who were taking a variety of selective serotonin reuptake inhibitors and serotonin noradrenaline reuptake inhibitors, and a female–male ratio that reflects the higher prevalence of MDD in females.^43^

Sampling might have been selective, favouring patients with previous negative discontinuation experiences, recurring episodes, and a longer total duration of antidepressant use. Eligible patients had to report a consideration or wish to discontinue their antidepressant, irrespective of the intensity of this wish. While this criterion may have been an important prerequisite for the formation of expectations regarding discontinuation, it may have favoured patients with more negative attitudes about antidepressants. We did not collect data on the number of treatment episodes, hospitalisation, or recourse to psychotherapy/counselling. Assessments of status and duration of remission, and number of episodes were based on self-report. In line with German guidelines, we defined remission as the presence of, at most, mild residual depressive symptoms. Patients with residual mild depressive symptoms may have more negative expectations about discontinuation. In our sample, residual depressive symptoms were identified as a barrier to discontinuation. However, we did not find relevant differences in expectations between the 26 patients with full remission versus the six patients with partial remission.

### Comparison with existing literature

To our knowledge, no other studies have examined expectations about antidepressant discontinuation. In this study patients indicated positive and negative expectations about discontinuation, including feeling their own identity again, increased or decreased quality of life, or being less resilient. Positive and negative expectations existed concurrently. Depending on the respective intensity, patients’ expectations increased or decreased the ambivalence between their wish to stop and their concerns about discontinuation. Positive expectations could facilitate, while negative expectations could hinder, discontinuation. Previous research has identified fears and confidence in ability to discontinue as barriers and facilitators.^32^^,^^33^ Authors had linked these categories to the concept of expectations, that is, anticipation of certain events such as recurrence, and recommended exploration of expectations before discontinuation.^33^ Our findings extend the existing body of research to include a comprehensive assessment of expectations, beyond fears and confidence.

Patients reported heterogeneous models about antidepressants’ mode of action and felt inadequately supported and informed about antidepressants in general, as well as timelines and methods for discontinuation. Participants’ discontinuation experiences had been predominantly negative and included recurrence and discontinuation symptoms. Their experiences with antidepressant use included not only effectiveness, but also side effects, psychological dependency, and further problems resulting from taking medication. Patients described balancing the risks and benefits of antidepressant use. If, over time, the risks outweighed the benefits, then patients were more inclined to attempt discontinuation. Our findings agree with those of a 2023 qualitative synthesis by Crowe *et al*,^36^ which analysed 27 studies on experiences of antidepressant use and discontinuation from mostly English-speaking Western countries. The authors summarised that, throughout treatment, patients tended to weigh the benefits of antidepressant use against the associated risks. Patients in the current study also reported a lack of information and guidance for discontinuation. Fear of recurrence and discontinuation symptoms lead to a vicious cycle, contributing to non-indicated long-term use of antidepressants.

Concerning barriers and facilitators, our findings in the German context are in line with findings from other Western countries.^10^^,^^32^^–^34^,^^44^ As also reported by Huijbers *et al*,^10^ in our sample, patients’ beliefs about MDD and antidepressants, such as that antidepressants would resolve a serotonin deficiency underlying MDD, affected their perceived need for continued antidepressant use. In line with previous research, we identified professional support and treatment information as being central facilitating factors and prerequisites for an informed decision about discontinuation.^28^^,^^34^^,^^35^^,^^37^ Our findings replicated recurrence, discontinuation symptoms, and further fears as being central barriers to discontinuation, often fuelled by previous negative experiences.^32^ We also found that such fears could be enhanced by reports within patients’ social environments, including the use of online forums, as pointed out during participant checking.^31^^,^^45^

Patients in our sample reported the wish to discontinue antidepressants within structured frameworks that provide information and professional support. The 2022 update of German treatment guidelines includes a significantly expanded section on discontinuation.^4^ It recommends information about treatment duration and possible difficulties arising from discontinuation early on, as well as close monitoring throughout the discontinuation process. However, this does not yet appear to have been implemented in routine practice.

### Implications for research and practice

Patients’ expectations can help or hinder antidepressant discontinuation. It is therefore important to promote functional expectations and take an individualised approach to minimise dysfunctional expectations towards discontinuation, adapted to patients’ previous experiences. Structured frameworks that provide patients with adequate information about treatment and professional support throughout discontinuation may help to optimise appropriate antidepressant use. Identified facilitators and barriers should be used to inform such frameworks. Further research, including with healthcare professionals, is required to inform design and implementation in routine practice. Future research could include patients who have successfully discontinued or are currently in the process of discontinuation; apply purposive sampling strategies with predefined ratios of negative versus positive previous experiences versus no previous discontinuation experience, and single versus recurrent episodes; and use a more extensive questionnaire battery combined with a clinical interview to assess remission based on self-report and expert judgement.
